# “Just lose weight”: weight-based medical bias and experiential expertise in intracranial hypertension

**DOI:** 10.3389/fpsyt.2026.1785195

**Published:** 2026-02-23

**Authors:** Kelly Moes

**Affiliations:** Centre for Culture and Technology, Curtin University, Perth, WA, Australia

**Keywords:** chronic illness, epistemic injustice, experiential expertise, idiopathic intracranial hypertension, medical bias, patient perspectives, weight stigma, weight-inclusive healthcare

## Abstract

**Introduction:**

Despite conflicting findings in the medical literature on Intracranial Hypertension (IH), weight gain is positioned as causative, and weight loss is regarded as "the only disease modifiable therapy." This paper explores the divergence between these medicalised framings of weight and the lived experiences of people with the condition, drawing on concepts such as epistemic injustice.

**Methods:**

The study draws on qualitative data from 563 adults with IH across 37 countries, generated through an online qualitative survey and an asynchronous digital research discussion group with 101 participants. Employing facet methodology and collective narrative enquiry within a critical disability studies framework, the research centres experiential expertise often overlooked in IH research and clinical practice.

**Results:**

Eighty-one per cent of participants reported being told that weight loss would cure their condition. Participants described healthcare encounters that placed excessive emphasis on weight at the expense of comprehensive investigations. Their experiential expertise challenges this emphasis: people with IH clearly contend that weight gain is a symptom rather than a cause, that weight loss frequently fails to deliver promised outcomes, and that hormonal and metabolic explanations make sense of their embodied experiences. The analysis shows how weight-centric approaches create an impossible bind, produce psychological and iatrogenic harm, and compromise health help-seeking behaviour. Weight-focused approaches also obscure the structural and social factors that independently contribute to IH outcomes.

**Discussion:**

Drawing on Mol's concept of the body multiple, the paper argues that clinical and experiential enactments of IH represent fundamentally different understandings of the condition, each incomplete without the other. Meaningful engagement requires recognising experiential expertise as a legitimate form of knowledge and adopting weight-inclusive approaches that prioritise symptom management and quality of life in IH research and practice.

## 1 Introduction

Intracranial Hypertension (IH) is a chronic illness characterised by dysregulated cerebrospinal fluid dynamics resulting in raised intracranial pressure. Primary symptoms include persistent severe headaches, visual disturbances (including potential vision loss), pulsatile tinnitus, and cognitive difficulties, although presentations vary widely among individuals. The condition predominantly affects women of reproductive age, with studies indicating that 70 to 90% of those diagnosed are classified within medical literature as having a BMI of 25 or over (1, 2). While considered rare, the incidence of IH has increased significantly in recent years. Population-based studies show a sixfold increase in prevalence, from 12 per 100,000 in 2003 to 76 per 100,000 in 2017, and a tripling in incidence, from 2.3 to 7.8 per 100,000 annually (3). Researchers have noted that these increases closely follow increases in average BMI at a population level, though they acknowledge the increase is multifactorial, including expanded MRI availability and increased diagnostic awareness (2, 3). Geographic and ethnic differences further complicate this picture, in which weight does not carry the same significance across diverse patient populations.

The condition carries various labels, including idiopathic intracranial hypertension (IIH), benign intracranial hypertension, secondary intracranial hypertension, and pseudotumor cerebri, many of which have been contested. This paper, therefore, uses ‘intracranial hypertension’ (IH) as a more inclusive term that encompasses all chronic forms of the condition and reflects the preferences of people living with IH. The term ‘idiopathic’ is especially significant, implying unknown causation even as biomedical discourse asserts weight as causative.

Despite an unknown aetiology and contradictory evidence in the literature about IH, the dominant medical narrative positions weight as the primary cause and weight loss as “the only disease modifiable therapy” (4, p. 478). Epidemiological association has directly translated into claims of causation and curative function (5), although weight is notably absent from the modified Dandy criteria used for diagnosis (6). In this study, 81% of participants reported being told that weight loss would ‘cure’ their condition (7, p. 178), reflecting how uncertain and mixed evidence has been translated into practice. Understanding the consequences of this requires a critical framework that examines medical authority and the exclusion of experiential expertise.

This paper employs a Critical Disability Studies (CDS) framework to examine how weight-focused clinical approaches shape experiences of IH. CDS extends beyond strict medical, legal, and identity-based definitions of disability, enabling analysis of experiences involving intersecting disabling factors such as chronic illness, medical trauma, and weight stigma (8). This intersectional approach is particularly useful for analysing IH, where multiple disabling experiences shape lived experience.

Central to this analysis is the medical model of disability, which locates the ‘problem’ within individual bodies rather than in social structures or environmental factors (9). As Brandon and Pritchard (10) argue, fatness and disability are complex social phenomena sharing similar experiences of discrimination and prejudice, requiring cross-theorising to understand how medical authority shapes both. Thomas (11, 12) extends this critique to chronic illness, highlighting how social deviance paradigms create particular challenges for people whose conditions lack clear biomedical explanations, requiring them to constantly negotiate their legitimacy. Wendell (13, p. 106) argues that cultures of individual responsibility for illness discourage investigation of social and environmental factors, instead locating the ‘problem’ within individual bodies. Within this framework, the person with IH is held responsible due to presumed failure to manage weight, and weight loss is positioned as the logical intervention. Critical fat studies scholarship highlights how the construction of fatness as pathology shapes health discourse and management of health conditions (14, 15). Healthcare settings have been identified as key areas of weight-based discrimination (16, 17). For people with IH (pwIH), this intersects with the condition’s clinical profile, where weight-based assumptions embedded in diagnostic and treatment pathways may lead to an oversight of comprehensive investigation.

The paucity of qualitative inquiry in IH research compounds these dynamics. Medicalised structures privilege biomedical knowledge and systematically devalue experiential expertise (18, 19). Lehman (20) identified that, despite ongoing efforts to describe IH, patient perspectives are not prioritised, and the impact of medical advice is rarely examined. Over twenty years later, this gap persists. Patient perspectives remain largely excluded from research and knowledge production (21). People with IH consistently report that their experiences are poorly represented in current medical interpretations and frequently encounter healthcare providers with limited knowledge of their condition (22). The consequences of this exclusion are evident in divergent research priorities. The IH Research Priority Setting Partnership found that healthcare providers prioritised weight and surgical interventions, while patients emphasised understanding causation, improving quality of life, and developing more effective treatments (23). Participants in this study express similar perspectives, articulating experiences that challenge medicalised assumptions about causation and cure:

“Our weight isn’t the answer. More of a question. With such a wide variety of symptoms and comorbid conditions I have seen discussed in the groups I am in, I’m thinking that IIH is not idiopathic, and is maybe just a symptom of other disorders.”

This insight inverts the clinical framing of IH, the divergence reflecting the gap between such experiential perspectives and the dominant medical narrative, which reflects whose knowledge is valued in shaping the research agenda.

This paper addresses that gap by centring the experiential knowledge of people living with IH. Drawing on collective narratives from 563 participants worldwide, it examines how the relationship between IH and weight is constructed within medical discourse, and how this influences the lived experience. I contend that this framing produces significant harms for people living with the condition. Following Kafer’s (24, p. 7) call not to dismiss medical approaches but to subject them to ‘renewed interrogation,’ the paper examines how weight-centred frameworks shape healthcare encounters, and result in inadequate treatment, psychological and iatrogenic harms, and changes in health help-seeking behaviour. Following critical fat studies scholarship, this paper uses ‘fat’ as a neutral descriptor of body size and refers to higher weight and BMI only when engaging and critiquing medicalised framings present in the IH literature. The paper argues for weight-inclusive approaches that prioritise the meaningful engagement of experiential expertise in IH research and practice.

## 2 Methodology

### 2.1 Study overview and design

This article draws on findings from doctoral research centring the perspectives of people living with IH (7). The research positions people who live with IH as experiential experts, whose embodied knowledge of the condition constitutes legitimate expertise. Following Couser (25), the study sought to move beyond the expression of illness experiences to actively reclaim the lived experience from medicalisation. The central research question asked, ‘In what ways does the lived experience of the IH community expand/or complicate the existing medical model of IH?.

The study adopted a facet methodology approach (26), recognising that the IH experience is “not only lived and experienced but is multi-dimensional, contingent, relationally implicated and entwined.” Situated within critical disability studies, the research employed ‘disability as method’ principles (27, 28) to intentionally incorporate the lived reality of chronic illness into the research design, enabling first-person perspectives to critically analyse the systems and practices embedded within IH’s medicalised meta-narrative.

Data generation employed two complementary methods. A qualitative survey conducted via Qualtrics in 2022 included 16 open-ended questions designed to gather narratives about lived experience, identity, and knowledge. Questions focused on social and cultural aspects of the IH experience, intentionally avoiding medicalised framing and addressing the research gap in personal and environmental factors identified by Lehman (20). Participants were recruited from 40 IH-specific Facebook groups and were required to be adults (18 years or over) with, or seeking, an IH diagnosis. The survey engaged 563 participants across 37 countries, including 16% from non-English-speaking backgrounds, with participants ranging from recently diagnosed to more than 10 years post-diagnosis.

The primary method of inquiry was an asynchronous, Facebook-mediated research discussion group (‘IH Voices’), with 101 participants drawn from survey respondents. This approach deliberately replicated the online knowledge-sharing spaces where the IH community already gathers. The asynchronous format accommodated crip-temporalities and energy limitations characteristic of this population, aligning with scholarship on online methods for geographically dispersed chronic illness communities (29–31). Participants were engaged as knowledge-holders rather than research subjects, with discussion topics shaped by their contributions and priorities. **Table 1** summarises participant characteristics across both phases of data collection.

**Table 1:** Summary of participant characteristics.

Characteristic	Qualitative survey (n=563)	Discussion group (n=101)
Gender	96% female	96% female
Age Range	18 to 65+ years (peak 30-40)	Representative across ranges
Countries Represented	37	17
Non-English primary language	16% (n=87)	12% (n=12)
Cultural/Ethnic Identity	75% White/European 25% other backgrounds, including First Nations (n=16), Black (n=12), Mixed Race (n=12)	N/A
Time since Diagnosis	42% (n=234) newly diagnosed (<4 years); 29% (n=155) mid-journey (5-9years); 27% (n=145) experienced (10+ years)	Approx. one-third in each category

Detailed participant demographics are available in Moes (7, Chapter 5).

The study’s focus on social, environmental and relational aspects of the lived experience meant that survey questions intentionally avoided weight-based narratives. Despite this, two-thirds of respondents raised weight unprompted in their open-ended responses, signalling its salience in shaping their experience. This participant-led emphasis informed subsequent group discussions about how profoundly this dominant narrative impacts the lived experience of IH.

Analysis employed collective narrative enquiry to identify shared patterns across individual accounts, attending to diversity of experience and the collective understanding. The sample and data size were guided by the notion of information power and “being greedy in the search for data, knowledge and insight” (26, p. 83; 32). The large and geographically diverse participant group enabled examination of whether IH experiences varied across healthcare systems and socio-cultural contexts. Notably, despite this diversity, the study found remarkable consistency in lived experience, countering assumptions that disability is too heterogeneous to support collective understanding.

Miranda Fricker (33) concept of epistemic injustice frames the analysis of participant accounts, attending to how their knowledge is received, dismissed or devalued within healthcare encounters. Testimonial injustice occurs when a speaker’s credibility is undermined due to prejudice. For pwIH, this is experienced when their narratives are dismissed or doubted due to weight-based or other medicalised assumptions. Hermeneutical injustice arises when interpretive resources fail to help make sense of their experiences. For those with IH, the dominance of the weight-based narrative leaves little space for alternative understandings of their condition. They counter this by participating in a large, global community of practice on social media that develops their experiential expertise (7, Chapter 5).

### 2.2 Ethics and researcher positioning

This study received ethical approval from Curtin University (HRE2022-0037). All participants provided informed consent. Survey participants could remain anonymous, while discussion group members could opt to be acknowledged by name as experts. Due to the sensitive nature of health experiences, particular care was taken to support participants’ well-being throughout the research process, reflecting the ‘disability as method’ principles embedded in the study design. Participants were provided with a comprehensive participation guide that included contact details for local mental health support services and direct contact information for the researcher. Content warnings were provided for all discussion topics, and participants were encouraged to engage only with topics they wished to contribute to. The sequencing of topics was also intentional, with emotionally demanding topics (such as weight) followed by lighter discussion. Regular check-ins with group members were conducted throughout the discussion to monitor and support members’ well-being.

Research quality was addressed through practices aligned with critical qualitative inquiry. Reflexive journaling supported ongoing examination of assumptions and interpretive choices. Check-ins with participants throughout the analysis ensured that interpretations accurately reflected their perspectives and incorporated alternative viewpoints. Extensive use of participants’ voices in reporting allows readers to evaluate the relationship between data and interpretation. All participant quotes are italicised throughout.

The researcher is a woman of larger body size who lives with IH and has been an active member of the IH community on Facebook for eight years. This emic positioning is methodologically aligned with the study’s approach. This insider position enabled access to community knowledge, built rapport and trust with participants, and supported deeper engagement with participant narratives and the nuanced interpretation of the data (34). The potential for over-identification was addressed through reflexive journaling, regular participant check-ins to test interpretations, and supervisory discussions throughout the research process. This approach aligns with crip-authorship principles (35), which position disabled people as authorities on their own experiences. This disability justice approach troubles the medicalised narrative by centring experiential knowledge that is systematically excluded from IH research and pushing back against the epistemic oppression that positions experiential expertise as less-than.

## 3 Analysis

A dominant medicalised narrative presents weight as both the cause of IH and the pathway to its cure, shaping how the condition is understood, researched, and treated. However, this confident position is at odds with a body of medical evidence that complicates, and sometimes directly contradicts, the weight-causation hypothesis. Participants’ experiential knowledge aligns more closely with this contradictory evidence than with the certainties that guide clinical practice. When the promised cure does not materialise, the consequences extend beyond physical symptoms to epistemic harm and the systematic devaluation of patient knowledge.

### 3.1 The weight meta-narrative

The biomedical narrative surrounding IH consistently presents weight as a primary causal factor, directly shaping clinical practice, where epidemiological correlation is readily mistaken for individual causation. Participants described their doctors as hyper-focused on weight, with clinical encounters often reduced to discussions of body size regardless of presenting concerns. This reflects the broader medicalisation of fatness, where higher bodyweight is viewed as pathological and weight loss as treatment. Such medicalisation is underpinned by what Metzl and Kirkland (36) term ‘the new health morality’, a neoliberal discourse that positions health as individual responsibility and places moral burden on people to discipline their bodies accordingly. Within this framework, fat bodies become symbolic of moral failure and, in line with the clinical gaze, produce a hierarchy of bodies in which those deemed fat are placed at the bottom (37–39).

In IH specifically, this is experienced as “synecdochical stigma” (40) whereby the whole person becomes reduced to their body size. BMI functions as both a diagnostic marker and treatment determinant, yet the measure itself is riddled with gender and racial bias (41). This centrality in IH classification reduces complex presentations to a single metric, blaming health conditions on weight while ignoring environmental and systemic factors. When a person with a stigmatised body encounters the clinical gaze, they encounter invalidation through treatment decisions shaped by weight bias and the dismissal of patient knowledge that contradicts weight-focused assumptions.

The persistence of weight-focused treatment despite contradictory evidence reflects the normalisation of weight bias in clinical practice. Just as disability scholars have shown how medical authority shapes understandings of disability (11), fat studies scholars have demonstrated how medical discourse constructs fatness as inherently pathological (14, 15, 42). In IH, these frameworks converge: weight-focused treatments are prescribed regardless of individual circumstances or contradictory evidence, and epistemic selectivity systematically undervalues experiential knowledge while clinical assumptions remain unchallenged.

### 3.2 Contesting causation

Challenging the weight-causation hypothesis requires engaging with both the contradictions within the medical literature and the experiential knowledge of people living with IH. Together, these forms of evidence align to challenge dominant assumptions and offer alternative explanatory frameworks. Treating correlation as causation has led to potentially harmful practices across healthcare (43). In IH, this association translates into clinical certainty that shapes both diagnosis and treatment.

While dominant discourse frames weight gain as causative and treatments subsequently prioritise weight loss, significant contradictory evidence exists within the research literature. While most pwIH have a BMI of 30 or above, many women of larger bodysize do not develop IH, and some individuals develop the condition with a BMI between 18 and 25 (44, 45). Even within the medical literature, language remains cautious: incidence rates are said to have ‘mirrored’ and ‘risen in parallel with’ (46), ‘correspond to’ (3), and ‘correlate with’ (47) population-level BMI trends. This is the language of association, not causation. Yet this cautious research language has been translated into confident clinical practice, with epidemiological associations reinforced and eventually treated as fact, even as proponents of weight-focused treatment acknowledge that the weight threshold for developing IH is unclear and weight is not a reliable indicator of visceral fat (48). Participants recognise this problem, and while they do not deny the importance of weight for overall health, with many acknowledging the general benefits of weight management and physical activity, they challenge the simplistic framing of weight as the cause and reliance on weight loss as the primary treatment, despite the evidence for this approach remaining uncertain:

“They need to look at the reasons as to why we have it and not just put it down to being overweight. I believe being overweight is a symptom, not a cause, and when more research is done, they will too.”

Geographic and ethnic differences further challenge the weight-causation hypothesis. If weight were simply causative, we would expect uniform patterns across populations; instead, BMI of 30 or over appears less common in Asian clinical groups and is rarely the primary risk factor in the Indian subcontinent (49–51).

Participants’ observations about temporal patterns were especially significant. The medical literature describes weight gain in the twelve months before diagnosis as a ‘risk factor’ supporting causation. However, participants interpreted this same observation differently:

“I gained a lot of weight before my IIH diagnosis because I was so unwell I couldn’t be very physically active. I attribute my weight gain to my IIH, rather than the other way around.”

This represents a fundamental disagreement about the same observed phenomenon. Medicine and patients agree that weight gain often precedes diagnosis; they differ in their interpretations. The biomedical narrative reads this as causal evidence, while experiential knowledge suggests weight gain may itself be symptomatic of underlying dysfunction. Significantly, this experiential interpretation is supported by the IH literature. Hannerz and Ericson (52) found that body size was not the cause of IH, suggesting instead that raised intracranial pressure may be the primary driver of weight increase, with higher body weight acting as a secondary factor in pre-existing IH. This inversion of the assumed causal direction aligns closely with participant accounts. Participants consistently reported weight gain occurring alongside other symptoms or developing quickly after diagnosis, with many noting they had a BMI of 25 or under prior to symptom onset. As participants reflected,

“I am more convinced … that weight is a metabolic symptom in MOST patients, not a cause for IIH.”

“Weight gain can be a symptom rather than a cause. That is my case and the case of many others I’ve spoken to. Shift focus from weight causing it and explore how the disease may trigger the weight gain.”

Participants also identified how the condition creates barriers to the very intervention prescribed as treatment:

“I am trying to lose weight, but when all I can do is get up and try to manage my pain, when even existing is exhausting … I haven’t got the resources to lose weight.”

The medical literature reflects similar uncertainty about weight loss as treatment. BMI at presentation does not appear to influence long-term visual or headache outcomes, with patients with BMI between 18 and 25 having outcomes similar to those with higher body weight (45). The only randomised controlled trial comparing bariatric surgery with community weight management found no statistically significant differences in IH-specific outcomes between patients who achieved significant weight loss (23kgs) to those with minimal weight loss (2kgs) (47). As Friedman (53, p. 678) observed, this raises fundamental questions about whether weight loss itself is the therapeutic mechanism. A recent systematic review of nine bariatric surgery studies reached a similar conclusion, acknowledged that findings “should be interpreted with caution given the single-arm nature of the available evidence and the predominance of observational studies” (54). Further complicating the picture, GLP-1 receptor agonists produce clinical benefits independent of BMI reduction (55), with ICP decreasing within hours before any weight change occurs (56, 57). Research also shows the same biological pathways (adipokines, inflammation, hormonal imbalance) are used to explain both how higher weight could cause IH and how rapid weight loss might trigger it (58), while cardiovascular risk in IH is noted as being independent and unrelated to higher body weight (59).

Participant accounts consistently pointed to alternative explanations, particularly hormonal and metabolic mechanisms. Demonstrating nuanced engagement with the medical literature, one participant explained,

“IIH can cause empty sella or partially empty sella syndrome. This is your pituitary gland being compressed by the high pressure, which then causes low pituitary hormones. Add to that, most of us have adrenal dysfunction and often thyroid problems.”

Another observed that weight is closely linked to hormonal influences, finding this association much more significant than weight alone. These experiential insights align with emerging research findings. Significantly elevated cerebrospinal fluid (CSF) leptin levels have been observed in pwIH, levels that do not correlate with BMI, suggesting hypothalamic dysfunction rather than adiposity as the mechanism. (60). Indeed, emerging medical research increasingly frames IH as a systemic metabolic disease with an evolving aetiology driven by metabolic dysregulation, involving a complex interplay of androgen excess, glucocorticoid dysfunction, and inflammation that affects CSF dynamics (44). This shift towards metabolic explanations aligns more closely with experiential knowledge than the reductive weight-causation narrative dominating clinical practice. As Potter et al. (44) acknowledge, “it is unknown what all the risk factors are that precipitate the condition” and “targeting certain hardwired risk factors is unachievable.” It is researchers rather than patients who are increasingly recognising that weight may be a factor in the disease, but not necessarily the cause.

Medical bias and the assumed profile of the ‘typical’ IH patient further complicate access to diagnosis and care. Medical literature consistently describes the typical patient as female, of childbearing age, with a BMI of 30 or over. This creates a paradoxical diagnostic situation where young women of larger body size may be prematurely diagnosed based on demographic profile, while those who do not fit the assumed profile may have their condition overlooked. This notion is not lost on pwIH who contend, *“They are so focused on overweight child bearing age women that they miss anyone outside this box.”* Participants at lower weights reported diagnostic dismissal or delay because they did not match clinical expectations: *“I have been told numerous times by different doctors I don’t look like I have IIH. I also have PCOS. I get told the same thing about that.”* Male participants also consistently report similar experiences, their symptoms dismissed because they did not match the expected profile. These accounts from those positioned as ‘atypical’ suggest the condition is more complex than weight-focused models allow.

### 3.3 Promise vs. reality – the curative fallacy and its consequences

“Either my body betrayed me, or the doctors did.”

This participant statement captures the central tension in experiential accounts. The medicalised promise is clear, lose weight and you won’t have any further problems. When that promise fails to materialise, those living with IH must navigate the gap between clinical expectations and everyday reality.

Weight was the most frequently mentioned issue when participants were asked the ‘one thing’ they wanted doctors or researchers to know, with one-third of respondents highlighting this concern. Many participants associate the emphasis on weight loss with inadequate treatment, believing their medical care has been undermined by the assumption that weight loss alone will resolve their symptoms.

While biomedical literature suggests that weight loss of 6-10% often leads to remission (61), participants report markedly different outcomes:

“We didn’t cause this because we are fat or lazy. We work hard and endure so much. Of course, every medical condition would improve with weight loss. I don’t believe that is the cure. I lost 60 lbs. It didn’t get any better. I was heartbroken.”

“If I could control it, I would. If weight loss would truly cure it what is the reason weight loss hasn’t helped many of us … Losing weight isn’t always the answer.”

The persistence of symptoms despite weight loss highlights what participants see as a key limitation of focusing solely on weight, *“weight is not a magical solution … Pushing weight loss risks overlooking other health concerns.”* Notably, participants also report their symptoms worsening after bariatric surgery, the very intervention increasingly promoted as a definitive treatment.

Some participants recognised broader health benefits from lifestyle changes while differentiating these from IH-specific outcomes. As one reflected, *“Being diagnosed with IIH has prompted me to change to a healthier lifestyle and I feel better for it but the symptoms didn’t go away.”* This nuanced perspective acknowledges the general value of healthy behaviours while contesting their positioning as curative for IH.

The failure of weight loss to deliver promised outcomes reflects broader patterns in literature on higher body weight. Long-term sustainability of weight-loss interventions remains poor, with research showing that weight lost in the first year is often regained, returning to baseline or above within 5 years (62–64). The health effects commonly attributed to fatness may be caused by weight-cycling itself, which has been associated with higher mortality, negative cardiovascular effects, and depression (63). This pattern is reflected in the IH literature. For example, Miah et al. (3) found that average BMI increased after diagnosis despite weight loss being the primary management strategy; however, less than 0.3% of their cohort had undergone bariatric surgery, whereas 9% had undergone CSF shunt procedures. Even when bariatric surgery is pursued, a recent systematic review found that up to 50% of patients experience primary weight loss failure or substantial weight regain (54). The current treatment approach recommends an intervention that most people cannot sustain, while surgical or pharmaceutical options targeting weight remain more commonly used than those addressing the impairment effects, making the prescribed intervention potentially counterproductive. The lived experience echoes these patterns, demonstrating the impact when the promised cure fails to materialise:

“I did a gastric sleeve surgery as per my doctor’s order and I lost 60 kilograms … after losing all this weight my condition has literally gotten a million times worse and I regret this decision so much.”

The psychological consequences of weight-focused care extend beyond frustration with inadequate treatment to produce measurable iatrogenic effects. These harms are interconnected: the paradigm framing weight as cause and cure generates psychological distress, which produces embodied health consequences that the same paradigm then attributes to patient behaviour.

Participants describe internalising messages about personal responsibility, experiencing shame and self-blame when weight loss doesn’t achieve expected results. The impossible bind operates not only in clinical encounters but in patients’ relationships with their own bodies:

“I feel like a failure. I’ve done everything they told me to do and I’m still sick. So either the treatment doesn’t work or there’s something wrong with me.”

“The guilt is overwhelming. Every time my symptoms flare I think, if only I’d tried harder, lost more weight, been more disciplined.”

Weight cycling, the repeated cycle of weight loss and regain, is associated with adverse health outcomes (63–65). If healthcare professionals recommend weight loss, patients attempt it, regain weight as research predicts most will, and are advised to lose weight again, this cycle itself may cause harm. Weight cycling, weight stigma, and healthcare inequality remain unexamined as confounding factors in IH research, yet these are known independent contributors to adverse health outcomes.

These narratives align with case evidence that further challenges the weight-causation narrative. Hamdan et al. (66) report a case of a man who developed IH after 60kg weight loss through diet and exercise. A previous case documented IH occurring during weight loss mediated by weight-loss drugs (67). These cases indicate that the link between weight and IH is more complex than the dominant narrative suggests, yet they are still treated as anomalies rather than prompting a re-evaluation of underlying assumptions.

The curative fallacy therefore operates on multiple levels, failing to deliver on promised outcomes, generating psychological harm through internalised blame and devaluation and dismissal in healthcare encounters, and may add further health consequences through weight cycling.

### 3.4 Epistemic injustice and the impossible bind

The disconnect between clinical emphasis on weight loss and participants’ lived experiences demonstrates systematic undervaluing of experiential knowledge. Participants confirm that their embodied experiences include a wider range of presentations than typically discussed, and that their symptoms are complex and interconnected rather than reducible to body size. Despite the consistency and sophistication of participant accounts, pwIH report that their experiences are often dismissed. Fricker’s (33) epistemic injustice framework illuminates these dynamics. Testimonial injustice occurs when patient reports of weight loss has not improved their symptoms are disregarded because they are viewed through the lens of weight stigma, compounded by the gendered demographics of IH. Hermeneutical injustice operates through the dominance of the weight-causation narrative, leaving those who understand their weight gain as symptomatic without the shared language to communicate this in healthcare encounters.

Participants recounted ongoing experiences of their knowledge being dismissed.

“Weight isn’t related. Different groups of people have IIH. Why do my neurological symptoms get so much worse when I don’t eat? That has nothing to do with weight.”

“I wish doctors understood that this isn’t our fault. We didn’t do this to ourselves. And losing weight won’t fix everything.”

This epistemic positioning creates practical consequences. The contradictions documented in medical literature create an impossible bind for patients. Do not lose weight and be blamed for IH persisting; lose weight ‘too slowly’ and be criticised for not trying hard enough; lose weight ‘too quickly’ and be blamed for triggering symptoms; maintain weight loss and still experience symptoms alongside collective disbelief. Regardless of patients’ actions, responsibility for treatment failure always lies with the individual rather than the limitations of the treatment approach.

This reflects what Grue (68) describes as the double bind faced by disabled people, where there are contradictory demands to overcome impairments through individual effort while having those efforts questioned; to accept medical authority yet advocate for their own needs; and to perform wellness while demonstrating sufficient impairment to access support. Fat studies scholars have identified a similar dynamic in the Good Fatty/Bad Fatty dichotomy (coined by Harding, 69, in Chastain, 70), where fat people are divided into those perceived as doing the “right” things (eating “correctly,” exercising, actively pursuing weight loss) and those perceived as failing to comply. “Good fatties” earn conditional tolerance through visible effort, while “bad fatties” are deemed deserving of stigma. For pwIH, these dichotomies intersect, where patients must continually demonstrate compliance with weight-loss regimens to be deemed deserving of care, yet the condition itself often renders this impossible. The dichotomy thus functions as a mechanism of social deviance, positioning those who cannot or do not lose weight as responsible for their ongoing illness.

Participants articulated this bind with clarity:

“I’ve been fat shamed and told that I need to lose weight to cure my IIH. I’ve also been told that losing weight too fast can cause IIH. So which is it?”

“They tell us to lose weight but the medications make us gain weight. The condition makes us exhausted so we can’t exercise. Then they blame us for not trying hard enough.”

The bind operates both epistemically and practically, limiting what patients can legitimately know and assert about their own bodies as well as what they can do. It turns a medical condition into a moral failing, compounding physical suffering with psychological harm. The impossible bind forces patients to locate failure somewhere, and the weight-focused approach ensures that place is never the approach itself. Further, participants who report that weight loss hasn’t improved their symptoms face disbelief, their experiential evidence dismissed as non-compliance or inadequate effort. Those who report worsening symptoms after weight loss are treated as anomalies.

### 3.5 Barriers to care and help-seeking

Healthcare encounters oriented toward thinner bodies exemplify Garland-Thomson’s (71) notion of “misfitting,” in which dissonance between body and environment creates spaces that are uncomfortable, unwelcoming, and unsafe (63). This misfitting is both material and intersubjective, shaping not only what care is offered but how patients are perceived and treated within those encounters. Participants consistently reported experiences of medical gaslighting, where symptoms are minimised, dismissed, or attributed to psychological causes. The collective experience expressed is that, regardless of the presenting complaint, weight becomes the default explanation: *“Every time you walk into the doctor’s, it doesn’t matter what you go in for, they always bring it back to your weight.”*.

This diagnostic overshadowing occurs in multiple directions. Participants with higher body weight report that all symptoms are attributed to body size, thereby delaying investigation of neurological issues, whereas those at lower body weight report diagnostic dismissal because they do not fit clinical expectations. Both patterns result in delayed diagnosis and treatment, with significant impacts on vision and quality of life. Body size, therefore, comes to represent the whole person, exemplifying synecdochic stigma, and obscures the complexity of individual presentations.

This is compounded by inadequate treatment, and healthcare professionals are often ill-equipped to support pwIH. Only 50% of participants reported receiving treatments that help with the day-to-day management of their condition (7, p. 177). As one participant stated, *“Nobody will offer me an adequate plan to address my pain or symptoms.”* Some participants have experienced treatment being withheld until they lose weight, prioritising body size change over symptom management and quality of life. Research by IIHUK confirms these patterns: among 625 respondents, 87% reported that they believed their condition was their fault due to their weight, while 78% were not offered support for weight management, and of those who were, 84% found it unhelpful or inappropriate (72, 73). The clinical encounter thus positions weight as the patient’s responsibility while failing to provide resources for the prescribed intervention and suggests that even weight-focused care often fails to meet patients’ needs.

The cumulative effect of these experiences influences not only the quality of healthcare encounters but also whether people seek healthcare at all. Participants described expecting negative encounters based on past experiences of weight stigma, leading some to delay or avoid seeking care altogether: *“I put off going to the doctor because I know what they’re going to say. It doesn’t matter what’s wrong, they’ll tell me to lose weight. So why bother?”*. Such avoidance carries specific risks for pwIH, where early intervention is significant for preserving vision. Weight-based discrimination leads to delayed care, cancelled appointments, and avoidance of health screenings (74, 75), while inadequate practitioner knowledge compounds these dynamics (7, 22, 76), potentially explaining the poor appointment adherence reported in IH (77).

Anticipated stigma also shapes how pwIH present themselves within healthcare settings. As Sheppard (78) notes, people with chronic illness often find themselves performing as ‘normal’ to avoid stigma while trying to appear ‘sick enough’ to be taken seriously. Zaks (79) explains how some learn to perform “the right kind of sick” to access necessary care. This is evident in the narratives of pwIH who describe self-censoring or minimising symptoms to avoid dismissive responses*, “I’ve stopped mentioning certain symptoms because I know they’ll just blame my weight. It’s easier to just deal with it myself.”* This strategic navigation adds burden while risking that clinicians receive incomplete information, compromising diagnostic accuracy and treatment planning.

Weight stigma in healthcare thus creates a self-perpetuating cycle. Negative experiences can lead to avoidance or guarded engagement, which may delay diagnosis or result in inadequate information exchange, thereby contributing to poorer outcomes. These outcomes are often attributed to patient non-compliance or the presumed health consequences of higher body weight. The weight-focused approach thus produces the very patterns it aims to address, while placing responsibility on individual patients rather than systemic bias. Participants described the cumulative toll:

“After years of being dismissed and blamed, you just lose trust. I don’t believe anything they tell me anymore because they’ve been wrong so many times.”

“It’s exhausting having to advocate for yourself every single time. Sometimes you just don’t have the energy to fight, so you accept whatever they say even when you know it’s not right.”

This erosion of trust has broader consequences beyond individual healthcare visits, influencing adherence to treatment advice, willingness to participate in research, and engagement with health services more generally.

### 3.6 Social determinants and intersecting inequities

The structural factors obscured by weight-focused approaches have key implications for IH. Population-level data demonstrates that over half of pwIH come from the two most deprived quintiles, and crucially, even after adjusting for BMI, significant associations between IH and deprivation remain in women. Miah et al. argue that socioeconomic deprivation itself, rather than body size in isolation, contributes to the underlying causes of IIHUK (73, p. e1256). These factors may include diet, pollution, smoking, and psychosocial stress, all of which have potential metabolic or endocrine effects. Cooper, Fernandes, and Cooper (80) argue that IH represents the visible tip of a much larger and expanding burden of health inequities that disproportionately affect women from lower socioeconomic backgrounds. This framing positions IH as a condition arising from broader patterns of structural inequality rather than from individual failures in weight management. Weight-focused clinical approaches, placing responsibility on individual patients, cannot address these upstream determinants.

Social determinants create compounding barriers to IH care. Women with IH are significantly more likely to delay seeking care due to rural residence, transportation difficulties, and inability to afford copays or specialist visits compared to women without IH (81). Socio-economic, racial, and gender disparities may be more pronounced in IH than when body size is considered in isolation, indicating that the intersection of chronic illness, weight stigma, and structural disadvantages produces compounded effects that weight-focused clinical approaches cannot address.

## 4 Discussion

*“Promoting weight loss as a treatment is actively harmful for many patients.”*.

The selective emphasis on weight in IH research and management reflects broader power dynamics in healthcare, where paternalistic assumptions position clinical expertise as more trustworthy than patient knowledge. Dewey’s concept of ‘selective emphasis’ describes how partial interpretations become viewed as complete explanations (82, pp. 25–30, cited in 83). Clinical interpretations are separated from their context to create abstractions that highlight certain aspects of a phenomenon while downplaying others, and normalising certain assumptions. In IH, the emphasis on weight as causative has become so dominant that alternative explanations, even those supported by evidence in the medical literature, struggle to gain acceptance. This selective emphasis obscures other IH-related factors that are independent of weight, including socioeconomic status, race, ethnicity, and gender. For conditions primarily affecting women, gendered patterns of medical dismissal intersect with weight stigma to compound epistemic disadvantage.

This selective emphasis leads to tangible harms for people living with IH. As Puhl and Heuer (16) contend, weight stigma is not a beneficial public health tool; rather, it threatens health, generates health disparities, and interferes with effective interventions. Perceived weight stigma doubles the likelihood of a ten-year allostatic load, raises mortality risk by 60%, and substantially heightens chances of metabolic syndrome, diabetes, and abdominal fatness (84). Body dissatisfaction alone predicts adverse health outcomes regardless of body size, with research showing that the gap between actual and preferred body weight is a stronger indicator of physical and mental health than BMI (85). The psychological experience of weight stigma may impact health more than body size itself.

Annemarie Mol’s (86) concept of ‘the body multiple’ offers a framework for understanding the deeper implications of this selective emphasis. Mol contends that disease is not a singular entity existing independently of practice but is enacted differently through various practices and perspectives. Applied to IH, this framework reveals how the condition is enacted differently across contexts. The clinical enactment views adiposity as causative and weight loss as curative, with treatment success measured by BMI reduction. The lived experience involves weight gain alongside symptom onset as part of broader bodily dysfunction, where weight loss often fails to resolve symptoms, and hormonal and metabolic explanations help make sense of embodied experience. These are not differences in perspective resolvable through better communication but fundamentally different understandings of what the condition is. When healthcare encounters are dominated by the medicalised perspective, any experiential evidence that complicates its assumptions are dismissed or devalued.

This framework explains the evident pattern in which medical researchers acknowledge the harms of weight stigma yet maintain weight-focused approaches. Mollan et al. (48, p. 8) recognise that higher rates of weight stigma among healthcare professionals, the public, and policymakers are associated with poorer patient outcomes, negative impacts on mental health, and avoidance of healthcare, noting barriers include a lack of sensitivity when discussing higher body weight and concerns about damaging the doctor-patient relationship. Despite this acknowledgement, weight-focused approaches remain the primary treatment.

When a medical system consistently locates the cause of illness in patient behaviour, the failure of prescribed interventions becomes personal failure. Cultural discourses that moralise weight and view higher body size as a character flaw compound this dynamic. Within this individualising perspective, weight gain becomes a matter of willpower and compliance rather than a complex phenomenon shaped by genetics, environment, medication effects, and the condition itself. Treatment failure is never viewed as a failure of the paradigm but always as patient failure. This framing operates within broader structures of harm and creates iatrogenesis through power imbalances within bureaucratic and paternalistic systems. The dominance of biomedical perspectives, combined with minimal meaningful engagement of pwIH, means social and environmental contexts remain underexplored, reinforcing patient narratives as the wrong kind of expertise. This constitutes systemic disablism, where pwIH face barriers created by epistemic selectivity and medical bias (7, Section 8.5). As one participant contended: *“Until the medical field changes their mind, we will continue to be marginalised”.*

Weight bias can also compromise diagnostic accuracy, with misdiagnosis or overdiagnosis rates in IH as high as 40% (87, 88). Young women with higher body weight may be diagnosed based on demographic profile rather than clinical evidence, while those who do not fit this profile may be missed entirely (89). Weight bias thus causes harm, as overdiagnosis subjects individuals to unnecessary interventions, whereas missed diagnoses delay treatment for those whose presentations do not match expectations.

Mental health issues are a well-documented comorbidity in IH. Over 70% of patients demonstrate “mild to moderate depression, anxiety and somatisation” (90), and appetite and eating disorders have been noted as high as 76% (91). These findings are typically interpreted as complicating IH management, with recommendations for early screening and multidisciplinary care. However, the direction of causality may warrant further investigation. The psychological distress documented in the IH literature (92–96) may also be partly iatrogenic, arising from the treatment paradigm rather than from IH itself. Stevens (38) demonstrates how discourses of health morality shape illness experience, finding that symptoms are not interpreted as illness when they serve the moral project of weight loss. When IH is framed as a consequence of weight, and weight as a moral failing, the repeated experience of being blamed for one’s condition may cause or worsen psychological distress.

Participants also expressed concerns that the hyper-focus on weight loss could be directly harmful, especially highlighting the overlooked needs of people with disordered eating and the pressure to undergo bariatric surgery. The failure to consider individuals’ history of eating disorders was starkly illustrated by one participant:

“I’m finding it hard to swallow that my weight has anything to do with it as even with my weight loss, the flare ups are bad. I also have a long history with my eating disorder and anorexia, so [this] weight loss could easily become much, much more…”

When asked whether their eating disorder had ever been considered, they responded: *“None, whatsoever.”*

Any previous or developing disordered eating or body dysmorphia remains unaddressed despite the emphasis on weight loss. As another participant cautioned:

“Weight loss is not a magical solution. It should not be pushed as such. Pushing weight loss to the extent that it is done risks causing disordered eating in vulnerable individuals, and absolutely risks overlooking other health concerns related to sudden and rapid weight loss … which can and does cause harm in favour of simply losing weight.”

Clinical approaches that locate the problem in patient behaviour might therefore perpetuate the very outcomes they aim to prevent. When pwIH are made to feel that their condition results from their weight, they experience the internalised stigma that research links to negative health outcomes. The medicalised meta-narrative has important implications for how pwIH are engaged in research about their condition and whether their perspectives are valued in its management. When experiential knowledge consistently indicates different understandings than clinical assumptions, the question becomes: whose enactment of the condition should influence treatment? The findings of this study suggest that meaningful engagement with experiential expertise could contribute to more effective and less harmful approaches to IH care.

### 4.1 Implications and future directions

The harms identified in this study are not accidental outcomes of weight-focused IH care but flow from its underlying assumptions. Meaningful change requires examining both clinical practices and the research frameworks that shape them, as well as wider cultural narratives that moralise weight and place responsibility for illness on individual patients.

These findings should be understood within the study’s limitations. Participants were a self-selected sample from English-speaking online communities who, although geographically diverse, were predominantly white, Western, and cisgender women. This predominance is to be expected, given the demographics of IH more broadly, and may therefore reflect the diagnostic bias in IH. These limitations are shared by much epidemiological research informing IH treatment, for example Kilgore et al. (46, p. 4) acknowledge that with 90% of their study population identifying as Caucasian, “it may be difficult to extrapolate these results to other non-Caucasian populations.” The researcher is a mid-sized fat woman living with IH who shares many of these characteristics. The collective narrative therefore primarily reflects white, Western perspectives, and the experiences of pwIH at intersections of multiple oppressions may differ significantly. Future research should investigate how weight-related experiences vary across healthcare systems and cultural settings, with particular focus on marginalised and non-English speaking communities.

Despite these limitations, this study demonstrates that pwIH hold valuable knowledge currently marginalised within clinical and research contexts. Participants offered sophisticated analyses of their symptoms, recognised patterns challenging mainstream explanations, and suggested alternative views based on embodied experience. This knowledge is not inferior to clinical knowledge but different in nature, offering insights biomedical methods cannot access. Systematic analysis of approximately 2,500 weight stigma research articles found that 64% paradoxically reinforce the pathologisation of fatness by portraying it as a disease or problem requiring elimination (97). Fox argues that much research on weight stigma is effectively research centred on higher body weight, and that patient perspectives are selectively included and framed in ways that reinforce pre-existing weight-reduction agendas. Research priorities should shift from focusing on weight loss to examining hormonal and metabolic processes, including leptin dysregulation and hypothalamic involvement. Experiential knowledge, rather than being dismissed as anecdotal, can play a meaningful role in forming hypotheses for scientific investigation.

In the context of IH, neither the medicalised conceptualisation nor the lived experience can offer a complete understanding without the other. Each represents only a fragment of the spectrum of IH; to fully understand the condition, each perspective requires the other. Moving forward, meaningful engagement of pwIH in research design, priority setting, and knowledge production is essential, recognising experiential expertise as a legitimate form of knowledge. Reframing IH as a complex, systemic condition with metabolic and hormonal components, rather than as a simple consequence of excess weight, would accommodate both clinical observations and experiential insights. This approach would not dismiss the association between IH and higher body weight but would contextualise it within a more nuanced understanding that does not reduce the condition to individual body size or locate responsibility for illness with patients.

Weight stigma functions as a ‘fundamental cause of (health) inequality,’ leading to health disparities through multiple pathways such as psychological distress, healthcare avoidance, diagnostic overshadowing, and inadequate medical treatment (84). The harms identified in this study flow from systemic frameworks, not just individual bias. As Westbury et al. (98) contend, solutions that focus solely on changing individual behaviours overlook the evidence supporting systemic and environmental interventions.

Clinical implications extend beyond individual healthcare providers. Weight-inclusive approaches to IH care should focus on symptom management and quality of life rather than body-size change, recognising that weight loss often fails to deliver expected outcomes. Participants emphasise the need for treatments that are holistic and centred on impairment effects and impact rather than each physical symptom in isolation. The Health at Every Size (HAES) framework offers one evidence-based model for such care, grounded in principles of weight inclusivity, health enhancement, respectful care, eating for well-being, and life-enhancing movement (99, 100). Research demonstrates that HAES approaches produce improved physical health outcomes independent of weight loss (101), suggesting that focusing on health behaviours rather than body size may be more effective for populations like those with IH. This calls for moving beyond fragmented specialties toward integrated care to address the multifaceted nature of IH. Meaningful change requires questioning assumptions embedded in clinical guidelines, research priorities, and medical education, as well as broader cultural narratives that moralise weight. The call from participants is clear:

“We live in our bodies and know when something seems off or different from our usual. Please just listen to us.”

## 5 Conclusion

This study centred the experiential knowledge of 563 people living with IH globally, examining how weight-based medical frameworks shape healthcare experiences and health outcomes. The analysis demonstrates that the dominant clinical narrative, which frames weight as both a cause and a cure, is experienced by many as a form of epistemic injustice that causes significant harm.

Participants’ experiential knowledge aligned not with confident clinical assertions but with contradictory evidence in the medical literature. They consistently understood weight gain as symptomatic rather than causative, identified barriers created by weight-focused treatment approaches, and articulated alternative explanatory frameworks centred on hormonal and metabolic dysfunction. The consequences of this disconnect extend beyond inadequate treatment to encompass psychological harm, iatrogenic effects, and changes in health help-seeking behaviour. Weight stigma operates not merely as a psychosocial burden but as a mechanism producing adverse health outcomes.

The findings contribute to growing scholarship that challenges weight-centric approaches to health and illness. For pwIH, the weight-focused treatment paradigm may be causing harm that is insufficiently recognised within clinical and research settings. Moving forward, it is essential to adopt epistemic humility regarding the weight-IH relationship, implement weight-inclusive approaches that prioritise symptom management and quality of life, and ensure meaningful engagement of pwIH in the production of knowledge about their condition. Meaningful engagement requires recognising clinical perspectives and experiential expertise as complementary forms of knowledge, each incomplete without the other. PwIH articulate this imperative clearly, and the final word is theirs:

“We need a balance between clinical-based knowledge and people living with a chronic illness knowledge.”

## Data Availability

The datasets presented in this study can be found in online repositories. The names of the repository/repositories and accession number(s) can be found below: https://espace.curtin.edu.au/handle/20.500.11937/98301.
